# Supplementary material for: Trends in low-density lipoprotein cholesterol goal achievement and changes in lipid-lowering therapy after incident atherosclerotic cardiovascular disease: Danish cohort study
Source: PLoS One. 2023 May 31;18(5):e0286376. doi: 10.1371/journal.pone.0286376 (PMC10231813; doi:10.1371/journal.pone.0286376)
Supplement: S1 File — (PDF) [file pone.0286376.s001.pdf]

S1 Table. Codes for diagnoses, procedures, and drugs.

Variable	Codes	Register and coding system
Acute myocardial infarction (AMI)	410* (ICD-8)<br>I21*-I23* (ICD-10)	The Danish National Patient Register (ICD codes)
Ischemic stroke (IS)	433*, 434*, 43601*, 43690* (ICD-8)<br>I63*, I64* (ICD-10)	
Unstable angina pectoris (UA)	411* (ICD-8)<br>I200* (ICD-10)	
Stable angina pectoris (SA)	413* (ICD-8)<br>I201*-I209 (ICD-10)	
Peripheral artery disease (PAD)	432*, 440*-445* (ICD-8)<br>I65*, I66*, I70*-I74*, I77* (ICD-10)	
Coronary angiography (CAG)	UXAC85	The Danish National Patient Register (procedure codes)
Computerized tomography coronary angiography (CT-CAG)	UXCC00A	
Coronary artery bypass grafting (CABG)	FNA*-FNE*, FNH20*	
Procedure of percutaneous coronary intervention (PCI)	FNG00-FNG12, FNG30	
Chronic kidney disease	581*-584* (ICD-8)<br>N02*-N08*, N11*-N14*, N158*-N160*, N162*-N165*, N168, N18*-N19*, Z940, Z992 E102, E112, E122, E132, E142, I12*-I13*, Q61*, M300, M313, M319, M321B, N26* (ICD-10)	The Danish National Patient Register (ICD codes)
	94300, 94340, 5748*<br>BJFD2*, KKAS*	The Danish National Patient Register (procedure codes)
Diabetes mellitus	249.00*-250.09* (ICD-8)<br>E10.0-E14.9* (ICD-10)	The Danish National Patient Register (ICD codes)
	A10A*, A10B*, C10AB04*	The Danish National Prescription Registry (ATC codes)
Statins	C10AA01- C10AA07	The Danish National Prescription Registry (ATC codes)
Ezetimibe	C10AX09	
Other non-statins<br>Fibrates<br>Bile acid sequestrants<br>Nicotinic acid and derivatives	C10AB01, C10AB02, C10AB04, C10AC01, C10AC02, C10AC04, C10AD06, C10AD52	
Combination therapy: ezetimibe with statins	C10BA02, C10BA05	

* Inclusion of all underlying codes.
ICD codes: International Classification of Diseases (ICD) versions 8 and 10.
ATC: Anatomical Therapeutic Chemical Classification System.

S2 Table. Distribution of low-density lipoprotein cholesterol (LDL-C) before and during hospitalization in individuals (N=15,396) with an incident (first ever) atherosclerotic cardiovascular disease (ASCVD), given by numbers.

		LDL-C during hospitalization			
		<1.8 mmol/L	≥1.8 mmol/L	None	Total
LDL-C before hospitalization	<1.8 mmol/L	367	224	955	1546
	≥1.8 mmol/L	350	4047	5460	9857
	None	138	2266	1589	3993
	Total	855	6537	8004	15,396

S3 Table. Development of low-density lipoprotein cholesterol (LDL-C) goal achievement in the atherosclerotic cardiovascular disease (ASCVD) population during the study period (1 January 2010 - 31 December 2015) given as proportion of individuals who reach treatment goals (LDL-C<1.8 mmol/L) within 1-180, 181-365 and 366-730 days after discharge, N=11,997 individuals.

**Incident ASCVD**		**LDL-C<1.8 mmol/L – days after discharge $N_{achieved}/N_{individuals}$ (% achieved)**					
**Year**	**N[a]**	**Achieved (days)**				**Not achieved <730 days**	**No LDL-C**
		**≤730**	**1-180**	**181-365**	**366-730**		
**2010**	2114	857/2114 (40.5%)	277/2114 (13.1%)	117/1967 (5.9%)	133/1902 (7.0%)	1257/2114 (59.5%)	330/2114 (15.6%)
**2011**	2142	881/2142 (41.1%)	358/2142 (16.7%)	146/1999 (7.3%)	129/1940 (6.6%)	1261/2142 (58.9%)	248/2142 (11.6%)
**2012**	2099	895/2099 (42.6%)	343/ 2099 (16.3%)	148/1943 (7.6%)	121/1891 (6.4%)	1204/2099 (57.4%)	283/2099 (13.5%)
**2013**	1935	849/1935 (43.9%)	334/1935 (17.3%)	150/1820 (8.2%)	141/1770 (8.0%)	1086/1935 (56.1%)	224/1935 (11.6%)
**2014**	1840	879/1840 (47.8%)	393/1840 (21.4%)	136/1716 (7.9%)	119/1660 (7.2%)	961/1840 (52.2%)	231/1840 (12.6%)
**2015**	1867	945/1867 (50.6%)	435/1867 (23.3%)	159/1773 (9.0%)	133/1725 (7.7%)	922/1867 (49.4%)	218/1867 (11.7%)
**p-value**		<0.001	<0.001	0.001	0.42	<0.001	0.003

[a] Individuals with incident atherosclerotic cardiovascular disease (ASCVD) who survived to discharge.

S4 Table. Development of lipid-lowering therapy (LLT) treatment patterns in the atherosclerotic cardiovascular disease (ASCVD) population during the study period (1 January 2010 - 31 December 2015) given as proportion of individuals who initiated treatment with (a) LLT and (b) intensive LLT before admission, from admission to 90 days after discharge, during 91-180 days after discharge and during 181-365 days after discharge, N=11,997 individuals with an incident ASCVD.

(a) Incident ASCVD		Initiation of moderate or intensive LLT treatment $N_{initiated}/N_{individuals}$ (% initiated)					
**Year**	**N**	**≤365 days**	**Before admission[a]**	**Admission to 90 after discharge**	**91-180 days after discharge**	**181-365 days after discharge**	**None**
**2010**	2114	1291/2114 (61.1%)	438/2114 (20.7%)	814/1676 (48.6%)	19/862 (2.2%)	20/843 (2.4%)	823/2114 (38.9%)
**2011**	2142	1298/2142 (60.6%)	437/2142 (20.4%)	825/1705 (48.4%)	19/880 (2.2%)	17/861 (2.0%)	844/2142 (39.4%)
**2012**	2099	1310/2099 (62.4%)	437/2099 (20.8%)	828/1662 (49.8%)	26/834 (3.1%)	19/808 (2.4%)	789/2099 (37.6%)
**2013**	1935	1190/1935 (61.5%)	431/1935 (22.3%)	707/1504 (47.0%)	28/797 (3.5%)	24/769 (3.1%)	745/1935 (38.5%)
**2014**	1840	1199/1840 (65.2%)	381/1840 (20.7%)	770/1459 (52.8%)	30/689 (4.4%)	18/659 (2.7%)	641/1840 (34.8%)
**2015**	1867	1245/1867 (66.7%)	359/1867 (19.2%)	845/1508 (56.0%)	26/663 (3.9%)	15/637 (2.4%)	622/1867 (33.3%)
**p-value**		<0.001	0.54	0.36	0.063	0.78	<0.001

(b) Incident ASCVD		Initiation of intensive LLT treatment $N_{initiated}/N_{individuals}$ (% initiated)					
**Year**	**N**	**≤365 days**	**Before admission[a]**	**Admission to 90 after discharge**	**91-180 days after discharge**	**181-365 days after discharge**	**None**
**2010**	2114	202/2114 (9.6%)	51/2114 (2.4%)	46/2063 (2.2%)	19/2017 (0.9%)	86/1998 (4.3%)	1912/2114 (90.4%)
**2011**	2142	196/2142 (9.2%)	47/2142 (2.2%)	64/2095 (3.1%)	16/2031 (0.8%)	69/2015 (3.4%)	1946/2142 (90.8%)
**2012**	2099	321/2099 (15.3%)	53/2099 (2.5%)	100/2046 (4.9%)	26/1946 (1.3%)	142/1920 (7.4%)	1778/2099 (84.7%)
**2013**	1935	359/1935 (18.6%)	64/1935 (3.3%)	116/1871 (6.2%)	31/1755 (1.8%)	148/1724 (8.6%)	1576/1935 (81.4%)
**2014**	1840	510/1840 (27.7%)	68/1840 (3.7%)	183/1772 (10.4%)	49/1589 (3.1%)	210/1540 (13.6%)	1330/1840 (72.3%)
**2015**	1867	612/1867 (32.8%)	67/1867 (3.6%)	226/1800 (12.6%)	56/1574 (3.6%)	263/1518 (17.3%)	1255/1867 (67.2%)
**p-value**		<0.001	<0.001	<0.001	<0.001	<0.001	<0.001

[a] At least one prescription redemption within 6 months before admission with a diagnosis of incident atherosclerotic cardiovascular disease (ASCVD).

S5 Table. Development of progressively more intensive lipid-lowering therapy (LLT) treatment in the atherosclerotic cardiovascular disease (ASCVD) population during the study period (1 January 2010 - 31 December 2015) given as proportion of individuals 180 days before admission with incident ASCVD to 90 days after discharge, 91-180 days and 181-365 days after discharge, N=11,997 individuals with an incident ASCVD.

Days in relation to ASCVD hospitali-zation	Total	Changes in LLT intensity				
		More intensive				
		Total	None → moderate	None or moderate → intensive	No changes	Less intensive [a]
				**Year of incident ASCVD event**		
				**2010**		
**-180 to 90 days**	2114	836 (39.5%)	790 (37.4%)	46 (2.2%)	1035 (49.0%)	243 (11.5%)
**91-180 days**	2007	157 (7.8%)	135 (6.7%)	22 (1.1%)	1405 (70.0%)	445 (22.2%)
**181-365 days**	1967	442 (22.5%)	310 (15.8%)	132 (6.7%)	1412 (71.8%)	113 (5.7%)
				**2011**		
**-180 to 90 days**	2142	845 (39.4%)	781 (36.5%)	64 (3.0%)	1043 (48.7%)	254 (11.9%)
**91-180 days**	2036	172 (8.4%)	155 (7.6%)	17 (0.8%)	1413 (69.4%)	451 (22.2%)
**181-365 days**	1999	420 (21.0%)	287 (14.4%)	133 (6.7%)	1474 (73.7%)	105 (5.3%)
				**2012**		
**-180 to 90 days**	2099	862 (41.1%)	762 (36.3%)	100 (4.8%)	1004 (47.8%)	233 (11.1%)
**91-180 days**	1978	161 (8.1%)	128 (6.5%)	33 (1.7%)	1365 (69.0%)	452 (22.9%)
**181-365 days**	1943	602 (31.0%)	269 (13.8%)	333 (17.1%)	1329 (68.4%)	123 (6.3%)
				**2013**		
**-180 to 90 days**	1935	742 (38.3%)	626 (32.4%)	116 (6.0%)	953 (49.3%)	240 (12.4%)
**91-180 days**	1849	155 (8.4%)	123 (6.7%)	32 (1.7%)	1294 (70.0%)	400 (21.6%)
**181-365 days**	1820	474 (26.0%)	222 (12.2%)	252 (13.9%)	1246 (68.5%)	100 (5.5%)
				**2014**		
**-180 to 90 days**	1840	804 (43.7%)	621 (33.8%)	183 (10.0%)	841 (45.7%)	195 (10.6%)
**91-180 days**	1752	159 (9.1%)	105 (6.0%)	54 (3.1%)	1108 (63.2%)	485 (27.7%)
**181-365 days**	1716	560 (32.6%)	205 (12.0%)	355 (20.7%)	1023 (59.6%)	133 (7.8%)
				**2015**		
**-180 to 90 days**	1867	894 (47.9%)	668 (35.8%)	226 (12.1%)	784 (42.0%)	189 (10.1%)
**91-180 days**	1796	168 (9.4 %)	107 (6.0%)	61 (3.4%)	1134 (63.1%)	494 (27.5%)
**181-365 days**	1773	616 (34.7%)	183 (10.3%)	433 (24.4%)	1029 (58.0%)	128 (7.2%)

Days in relation to ASCVD hospitali-zation	Total	Changes in LLT intensity				
		More intensive				
		Total	None → moderate	None or moderate → intensive	No changes	Less intensive [a]
		p-value				
**-180 to 90 days**		<0.001	0.008[b]	<0.001	<0.001	0.25[b]
**91-180 days**		0.071	0.34[b]	<0.001	<0.001	<0.001[b]
**181-365 days**		<0.001[b]	<0.001	<0.001	<0.001	0.010[b]

[a] Less intensive included change in lipid-lowering therapy (LLT) intensity from intensive to moderate or none, and from moderate to none.

[b] No linear trend.

S6 Table. Development of low-density lipoprotein cholesterol (LDL-C) goal achievement in the atherosclerotic cardiovascular disease (ASCVD) population during the study period (1 January 2010 - 31 December 2015) given as proportion of individuals who reach treatment goals (LDL-C<1.4 mmol/L) within 1-180, 181-365 and 366-730 days after discharge, N=13,125 individuals.

**Incident ASCVD**		**LDL-C<1.4 mmol/L – days after discharge** $N_{achieved}/N_{individuals}$ **(% achieved)**					
**Year**	**N**[a]	**Achieved (days)**				**Not achieved <730 days**	**No LDL-C**
		**≤730**	**1-180**	**181-365**	**366-730**		
**2010**	2329	616/2329 (26.4%)	115/2329 (4.9%)	57/2164 (2.6%)	76/2085 (3.6%)	1713/2329 (73.6%)	368/2329 (15.6%)
**2011**	2346	559/2346 (23.8%)	142/2346 (6.1%)	75/2177 (3.3%)	55/2110 (2.6%)	1787/2346 (76.2%)	287/2346 (11.6%)
**2012**	2269	577/2269 (25.4%)	144/2269 (6.4%)	74/2096 (3.5%)	51/2039 (2.5%)	1692/2269 (74.6%)	308/2269 (13.5%)
**2013**	2115	511/2115 (24.2%)	120/2115 (5.7%)	71/1978 (3.6%)	72/1922 (3.8%)	1604/2115 (75.8%)	248/2115 (11.6%)
**2014**	2015	587/2015 (29.1%)	188/2015 (9.3%)	73/1873 (3.9%)	70/1814 (3.9%)	1428/2015 (70.9%)	256/2015 (12.6%)
**2015**	2051	600/2051 (29.2%)	192/2051 (9.4%)	74/1941 (3.8%)	91/1888 (4.8%)	1451/2051 (70.8%)	243/2051 (11.7%)

[a] Individuals with incident atherosclerotic cardiovascular disease (ASCVD) who survived to discharge.

S7 Table. Sociodemographic and clinical characteristics associated with initiation of lipid-lowering therapy (LLT) (moderate or intensive) from admission to 90 days after discharge among individuals with incident atherosclerotic cardiovascular disease (ASCVD) and no LLT therapy before admission. Figures given are number of individuals ($N_{individuals}$), number and proportion of individuals with initiation of LLT therapy ($N_{initiated}$, %), odds ratio (OR) and 95% confidence interval (95% CI), N=9514 individuals with an incident ASCVD with no LLT therapy before admission.

		Initiation of LLT therapy (moderate or intensive) between admission and 90 days after discharge		
**Characteristics**	**$N_{individuals}$**	**$N_{initiated}$ (%)**	**OR[c]**	**95% CI**
**Qualifying ASCVD event at index date**				
**AMI**	2556	1862 (72.8%)	10.84	9.41; 12.48
**IS**	3001	2002 (66.7%)	8.77	7.65; 10.05
**UA+CAG**	277	122 (44.0%)	3.25	2.49; 4.23
**SA+CAG/CT-CAG**	2063	408 (19.8%)	1	ref
**PAD**	1065	257 (24.1%)	1.39	1.16; 1.66
**CABG**	208	37 (17.8%)	0.97	0.67; 1.41
**PCI**	344	101 (29.4%)	1.77	1.36; 2.29
**Age group (years)**				
**<40**	102	78 (76.5%)	3.95	2.48; 6.28
**40-49**	696	486 (69.8%)	2.85	2.38; 3.41
**50-59**	1711	946 (55.3%)	1.51	1.34; 1.71
**60-69**	2833	1313 (46.3%)	1.06	0.95; 1.18
**70-79**	2764	1241 (44.9%)	1	ref
**≥80**	1408	725 (51.5%)	1.31	1.15; 1.50
**Gender**				
**Male**	5964	3043 (51.0%)	1.05	0.97; 1.15
**Female**	3550	1746 (49.2%)	1	ref
**Cohabitation**				
**Yes**	3853	2065 (53.6%)	1	ref
**No**	5661	2724 (48.1%)	1.21	1.11; 1.31
**Ethnicity**				
**Denmark**	8948	4497 (50.3%)	1	ref
**Western**	248	135 (54.4%)	1.14	0.88; 1.48
**Non-western**	318	157 (49.4%)	0.81	0.65; 1.02
**Diabetes mellitus**				
**Yes**	1456	404 (27.7%)	1	ref
**No**	8058	4385 (54.4%)	3.12	2.75; 3.53
**Chronic kidney disease**				
**Yes**	300	93 (31.0%)	1	ref
**No**	9214	4696 (51.0%)	2.29	1.78; 2.94
**Socioeconomic position (quartiles based on income)[a]**				
**Q_1 (lowest 25%)**	2403	1195 (49.7%)	1	ref
**Q_2**	2328	1141 (49.0%)	0.95	0.85; 1.07
**Q_3**	2280	1160 (50.9%)	1.01	0.90; 1.13
**Q_4 (highest 25%)**	2503	1293 (51.7%)	1.07	0.95; 1.20
**LDL-C measurements within 18 months before ASCVD hospitalization[b]**				
**Yes**	7664	3113 (40.6%)	1	ref
**No**	1850	1676 (90.6%)	13.17	11.18; 15.51
**LDL-C measurement before or during ASCVD hospitalization**				
**<2.4**	2236	329 (14.7%)	1	ref
**2.4-3.8**	5086	2789 (54.8%)	6.99	6.13; 7.96
**>3.8**	2192	1671 (76.2%)	18.38	15.73; 21.46
**HDL-C measurement before or during ASCVD hospitalization**				
**<1.0**	1641	862 (52.5%)	1.01	0.88; 1.16
**1.0-1.5**	5666	2860 (50.5%)	1.02	0.92; 1.13

**>1.5**	2207	1067 (48.3%)	1	ref
**Total cholesterol (TC) measurement before or during ASCVD hospitalization**				
**<4.4**	2338	515 (22.0%)	1	ref
**4.4-5.9**	4827	2574 (53.3%)	4.14	3.69; 4.65
**>5.9**	2349	1700 (72.4%)	9.75	8.50; 11.18
**Triglycerides**				
**<1.0**	2167	1232 (56.9%)	1	ref
**1.0-2.0**	5009	2476 (49.4%)	0.74	0.67; 0.82
**>2.0**	2338	1081 (46.2%)	0.60	0.53; 0.67

ASCVD: Atherosclerotic cardiovascular disease; AMI: Acute Myocardial Infarction; UA: Unstable angina pectoris; CAG: Coronary angiography; SA: Stable angina pectoris; PCI: Percutaneous coronary intervention; CABG: Coronary artery bypass grafting; PAD: Peripheral artery disease; IS: Ischemic stroke.

[a] Socioeconomic position is derived as quartiles based on income. Quartiles are estimated for combinations of sex (male, female) and age (age<65 years, age≥65 years), for each year, separately.

[b] The latest available lipid measurement is identified up to 18 months prior to index date.

[c] Adjusted for age group and sex.

S8 Table. Development of lipid-lowering therapy (LLT) treatment patterns in the atherosclerotic cardiovascular disease (ASCVD) population during the study period (1 January 2010 - 31 December 2015) stratified by LLT intensity (moderate or intensive, intensive) and type of ASCVD diagnosis (acute myocardial infarction and ischemic stroke (AMI/IS) versus other) before admission, and from admission to 90 days after discharge, N=11,997 individuals.

Year	Initiation of LLT therapy, $N_{initiated}$ (% initiated)							
	Moderate or intensive LLT				Intensive LLT			
	Before admission[a]		Admission to 90 after discharge		Before admission[a]		Admission to 90 after discharge	
	AMI/IS	Other[b]	AMI/IS	Other[b]	AMI/IS	Other[b]	AMI/IS	Other[b]
**2010**	300 (23.7%)	138 (16.3%)	650 (67.4%)	164 (23.1%)	17 (1.3%)	34 (4.0%)	33 (2.6%)	13 (1.6%)
**2011**	266 (21.3%)	171 (19.1%)	683 (69.6%)	142 (19.6%)	15 (1.2%)	32 (3.6%)	52 (4.2%)	12 (1.4%)
**2012**	250 (21.3%)	187 (20.2%)	658 (71.2%)	170 (23.0%)	18 (1.5%)	35 (3.8%)	66 (5.7%)	34 (3.8%)
**2013**	247 (22.2%)	184 (22.4%)	556 (64.1%)	151 (23.7%)	27 (2.4%)	37 (4.5%)	89 (8.2%)	27 (3.4%)
**2014**	243 (21.7%)	138 (19.1%)	613 (70.1%)	157 (26.9%)	19 (1.7%)	49 (6.8%)	146 (13.3%)	37 (5.5%)
**2015**	245 (20.6%)	114 (16.8%)	704 (74.6%)	141 (25.0%)	17 (1.4%)	50 (7.4%)	182 (15.5%)	44 (7.0%)

AMI: Acute Myocardial Infarction; IS: Ischemic stroke.

[a] At least one prescription redemption within 6 months before admission with a diagnosis of incident atherosclerotic cardiovascular disease (ASCVD).

[b] Unstable angina pectoris (UA) with coronary angiography (CAG), stable angina pectoris (SA) with CAG/CT-CAG, peripheral artery disease (PAD), coronary artery bypass grafting (CABG), and percutaneous coronary intervention (PCI).
