# Supplementary material for: Trends in low-density lipoprotein cholesterol goal achievement and changes in lipid-lowering therapy after incident atherosclerotic cardiovascular disease: Danish cohort study
Source: PLoS One. 2023 May 31;18(5):e0286376. doi: 10.1371/journal.pone.0286376 (PMC10231813; doi:10.1371/journal.pone.0286376)
Supplement: Attachment — Submitted filename: RevisionNote.docx [file pone.0286376.s002.docx]

**PONE-D-21-35228**

Trends in low-density lipoprotein cholesterol goal achievement and changes in lipid-lowering therapy after incident atherosclerotic cardiovascular disease: Danish cohort study.

Deadline: Apr 08 2022

| **REVISION NOTE** | | |
| --- | --- | --- |
|  | | |
| **ADDITIONAL REQUIREMENTS** | **RESPONSE** | |
| 1. Please ensure that your manuscript meets PLOS ONE's style requirements, including those for file naming. | The manuscript has been revised according to the PLOS ONE’s style requirements. | |
| 2. Please include your tables as part of your main manuscript and remove the individual files. Please note that supplementary tables (should remain/ be uploaded) as separate "supporting information" files. | Tables are included as part of the revised manuscript and removed as individual files.  Supplementary tables are remained as separate tables. | |
| 3. Thank you for stating the following in the Funding Section of your manuscript:  “This work was supported by Sanofi Aventis Denmark A/S; Applied Economics and Health Research (ApHER).  This paper is based on data originating from a study conducted for Applied Economics and Health Research (ApHER) as an independent research institute and funded by Sanofi Aventis Denmark A/S.”  We note that you have provided additional information within the Funding Section that is not currently declared in your Funding Statement. Please note that funding information should not appear in other areas of your manuscript. We will only publish funding information present in the Funding Statement section of the online submission form.  Please remove any funding-related text from the manuscript and let us know how you would like to update your Funding Statement. Currently, your Funding Statement reads as follows:  “This work was supported by Sanofi Aventis Denmark A/S and Applied Economics and  Health Research (ApHER).  This paper is based on data originating from a study conducted for Applied Economics and Health Research (ApHER) as an independent research institute and funded by Sanofi Aventis Denmark A/S.  www.sanofi.dk  http://appliedeconomics.dk/  The sponsors (Sanofi) was involved in the Conceptualization and the final review and editing of the manuscript”  Please include your amended statements within your cover letter; we will change the online submission form on your behalf. | Funding section has been deleted in the revised manuscript.  We would the Funding Statement as follows:  “This work was supported by Sanofi Aventis Denmark A/S (www.sanofi.dk) and Applied Economics and Health Research (ApHER) (appliedeconomics.dk).  The paper is based on data originating from a study conducted for Applied Economics and Health Research (ApHER) as an independent research institute and funded by Sanofi Aventis Denmark A/S.  The sponsor (Sanofi) was involved in the conceptualization and the final review and editing of the manuscript”  This information is also described in the cover letter to the editor. | |
| 4.  Thank you for stating the following in the Competing Interests section:  “I have read the journal's policy and the authors of this manuscript have the following competing interests:  When this study was performed, KH Mikkelsen was an employee of Sanofi Aventis Denmark A/S  A Green and SM Hede were associated with ApHER that received funding from Sanofi Aventis Denmark A/S during the conduct of the study, and they declare no other relationships or activities outside the submitted work  GG declare that he has received funding from Bristol Myers Squibb, Bayer, Boehringer Ingelheim and Pfizer outside the submitted work.  All other authors declare no conflict of interest.”  Please confirm that this does not alter your adherence to all PLOS ONE policies on sharing data and materials, by including the following statement: "This does not alter our adherence to  PLOS ONE policies on sharing data and materials.” (as detailed online in our guide for authors http://journals.plos.org/plosone/s/competing-interests).  If there are restrictions on sharing of data and/or materials, please state these. Please note that we cannot proceed with consideration of your article until this information has been declared.  Please include your updated Competing Interests statement in your cover letter; we will change the online submission form on your behalf. | Competing Interests section has been deleted in the revised manuscript.  We would the Competing Interests Statement as follows:  “I have read the journal's policy and the authors of this manuscript have the following competing interests:  When this study was performed, KH Mikkelsen was an employee of Sanofi Aventis Denmark A/S  A Green and SM Hede were associated with ApHER that received funding from Sanofi Aventis Denmark A/S during the conduct of the study, and they declare no other relationships or activities outside the submitted work  GG declare that he has received funding from Bristol Myers Squibb, Bayer, Boehringer Ingelheim and Pfizer outside the submitted work.  All other authors declare no conflict of interest.  This does not alter our adherence to PLOS ONE policies on sharing data and materials.”  This information is also described in the cover letter to the editor. | |
|  | | |
| **ADDITIONAL EDITOR COMMENTS** | **RESPONSE** | |
| You are invited to consider the reviewers' comments, reported at the end of this letter, and to revise your manuscript accordingly. In the letter accompanying your resubmission, please explain your response to each of the comments. Please observe the word count and citation style. For further details, please consult the Instructions for Authors on the website. | Thank you for the comments to the editor and reviewers.  We have revised the manuscript accordingly and addressed all comments and suggestions.  This is described in detail in this point-to-point revision note, see below. | |
|  | | |
| **REVIEWER #1 COMMENTS TO THE AUTHOR** | **RESPONSE** | |
| The authors presented data to investigate trends in LDL-C goal achievement, initiation of lipid-lowering therapy and changes in LLT intensity in a population with atherosclerotic cardiovascular disease. The topic is of interest. The authors found that an increased proportion achieved LDL-C goal within the first 2 years after index date.  General comments:  1) While the paper is within the word limit, I feel that readability would be enhanced by being more succinct and shortening the word count. | Thank you very much for the comments and suggestions to the manuscript. We have revised the manuscript accordingly and addressed all comments in the point-to-point revision note, see below.  In order improve readability, we have revised the manuscript to be more concise and succinct.  The individual changes in the revised manuscript based on this comment are not shown in this revision note. | |
| Detailed comments:  2) Please describe the inclusion and exclusion criteria more in detail. | We have included a separate section with inclusion and exclusion criteria. Also, we have described in more detail all exclusion criteria applied in figure 1.  *The new section is as follows*:  **“Inclusion and exclusion criteria**  To be included in the study, individuals should be residents of Funen Island, Denmark, at index date. The population in the Region of Southern Denmark including Funen is a representative sample of the entire Danish population [26]. Furthermore, individuals should have at least one LDL-C measurement up to 18 months before index date or during ASCVD hospitalization (i.e., index date to date of discharge).  We excluded individuals with an ASCVD diagnosis in the period 1977-2009 to ascertain truly first-ever ASCVD cases. In case of two or more ASCVD events at the same date, we coded ASCVD in the following order: AMI, IS, UA, SA, PAD, and CABG. Individuals were also excluded if the unique personal identification number was invalid, if the address was outside the Funen municipality at date of the ASCVD diagnosis, or if they died during the ASCVD hospitalization. Finally, individuals were excluded if the LDL-C measurement during ASCVD hospitalization was <1.8 mmol/L (70 mg/dL) or no LDL-C measurements were available during or before ASCVD hospitalization.” | |
| 3) The authors consider LDL-C assays up to 18 months before the event. They do not believe this is a very large time frame for an assessment of the lipid profile? Please specify. | Individuals in the study population should have at least one LDL-C measurement before the qualifying event. An 18-month period before the qualifying event was applied to ensure a sufficient sample size. The measurement closest to the event date was used if more than one LDL-C measurement was available. | |
| 4) What definition of PAD did they use? | We used the following ICD-8 and ICD-10 codes to define PAD as a diagnosis in the Danish National Patient Register. This is described in the Supplementary Table S1  ICD-8: 432*, 440*-445*  ICD-10: I65*, I66*, I70*-I74*, I77*  where ”*” means inclusion of all underlying codes. | |
| 5) Dosing timepoints were different for different patients. This is an important limitation. Don't the authors think they should mention it? | We agree that the lack of prescribed dosage is an important limitation. We have mentioned this in the revision of the Limitations section.  The first part of the Limitations section is modified:  *from:*  “The study also has some limitations. First, we had no information on reasons for a suboptimal LLT, and no data on actual consumption of drugs as information on LLT use was based on prescription data.”  *to*:  “The study also has some limitations. The health registers do not contain data that might explain the patterns identified. Thus, we had no information on reasons for a suboptimal LLT. The available prescription data regarding LLT are based on prescription redemption but without data on actual issuing a prescription. Data include information on date and amount of drug prescribed, but do not inform on the specific dosage to be used by the individual user. Neither do we have information about the actual consumption of drugs.” | |
| 6) The authors mention "other non-statins" several times. What therapies are they referring to?  What is meant by "combination therapies"? Isn't ezetimibe already in combination with statins? Please specify this point. | Other non-statins are:  Fibrates: ATC: C10AB01, C10AB02, C10AB04  Bile acid sequestrants: ATC: C10AC01, C10AC02, C10AC04  Nicotinic acid and derivatives: ATC: C10AD06, C10AD52  The ATC codes are given in supplementary Table S1.  The therapy categories (fibrates, bile acid sequestrants, nicotinic acid and derivatives) are included in the revised version of Table S1.  Combination therapy refers to one tablet containing both ezetimibe and statins.  The two drugs (ATC: C10BA02, C10BA05) are combinations of ezetimibe with either simvastatin or atorvastatin, respectively.  *Table S1 is revised as follows*: | |
|  | Other non-statins  Fibrates  Bile acid sequestrants  Nicotinic acid and derivatives | C10AB01, C10AB02, C10AB04, C10AC01, C10AC02, C10AC04, C10AD06, C10AD52 |
|  | Combination therapy: ezetimibe with statins | C10BA02, C10BA05 |
| 7) The results seem to run counter to international data showing reduced adherence with respect to statin intake manifested by poor target attainment. How do the authors explain this difference? | A few previous studies have shown an improvement in LDL-C achievement and an increase in LLT initiation. Our results are in line with these previous findings, and we do not see any differences. However, despite this improvement, a large proportion of the individuals did not reach the LDL-C goal within 2 years after the ASCVD event. The health registers do not contain further data that may elucidate explanations and mechanisms underlying the findings and we have discussed the findings in this context.  We have *added a short statement* on the lack of explanatory data in the section Limitations as part of the Discussion. The first part of the Limitations section is modified:  *from:*  “The study also has some limitations. First, we had no information on reasons for a suboptimal LLT, and no data on actual consumption of drugs as information on LLT use was based on prescription data.”  *to*:  “The study also has some limitations. The health registers do not contain data that might explain the patterns identified. Thus, we had no information on reasons for a suboptimal LLT. The available prescription data regarding LLT are based on prescription redemption but without data on actual issuing a prescription. Data include information on date and amount of drug prescribed, but do not inform on the specific dosage to be used by the individual user. Neither do we have information about the actual consumption of drugs.” | |
| 8) There are errors in the calculation of some percentages in Table 1. Please recheck the section "Lipid lowering therapy within 180 days before ASCVD hospitalalization" and the " Intensity of lipid lowering therapy within 180 days before ASCVD hospitalization " section. | Thank you very much for bringing this error to us. For these sections, we have used the column-wise proportions rather than the row-wise proportions.  Figures in Table 1 in the section: “Lipid lowering therapy within 180 days before ASCVD hospitalization” are correct.  However, figures in Table 1 in the section “Intensity of lipid lowering therapy within 180 days before ASCVD hospitalization" and in the section “Comorbidity” are revised (highlighted by yellow). | |
| 9) The authors should at least mention new therapeutic strategies that allow the ambitious new targets to be reached. Reference is made to PCSK9 inhibitors and their effects on adherence and quality of life. (doi: 10.1056/NEJMoa1801174; 10.1056/NEJMoa1615664; 10.1016/j.phrs.2019.03.021; 10.1177/2047487319839179; 10.2459/JCM.0000000000000611) | The suggested references describe studies examining the effect of PCSK9 inhibitors on reducing risk of recurrent cardiovascular events compared with standard LLT. However, this is not the purpose of our study. We examined the development in the proportion of individuals initiating LLT and the proportions of individuals with LDL-C goal achievement in the period 2010-2015.  In the introduction, we have *included a sentence* describing, that new drugs are available including PCSK9 as follows:  “New drugs for managing dyslipidemia have recently emerged, such as proprotein convertase subtilisin/kexin type 9 (PCSK9) inhibitors [10,11].”  *Additional references are included*:  10. Sabatine MS, Giugliano RP, Keech AC, Honarpour N, Wiviott SD, Murphy SA, et al. for the FOURIER Steering Committee and Investigators. Evolocumab and Clinical Outcomes in Patients with Cardiovascular Disease. N Engl J Med 2017; 376:1713-1722.  11. Schwartz GG, Steg PG, Szarek M, Bhatt DL, Bittner VA, Diaz R, et al. for the ODYSSEY OUTCOMES Committees and Investigators. Alirocumab and Cardiovascular Outcomes after Acute Coronary Syndrome. N Engl J Med 2018; 379:2097-2107. | |
|  | | |
| **REVIEWER #2 COMMENTS TO THE AUTHOR** | **RESPONSE** | |
| The study analyses the proportions of high cardiovascular risk patients achieving the recommended therapeutic target for LDL-C after starting lipid-lowering therapy (LLT) following an acute cardiovascular event in a large population sample from Funen Island in Denmark. The manuscript is overall well written and the statistical analysis is properly conducted. In addition, the study includes a very large population sample, which render the main findings of the analysis of potential clinical relevance. Some methodological aspects, however, remain unclear to me and should be better defined. I have some comments and suggestions to propose: | Thank you very much for the comments and suggestions to the manuscript. We have revised the manuscript accordingly and addressed all comments in the point-to-point revision note, see below. | |
| #1. It would be very useful for the readers to have also the converted units (mg/dl), eventually between parentheses, for each value reported within the text. This would render the manuscript more easy to follow for those who are less confident with international units. | We have included the LDL-C levels in mg/dL as well as mmol/L.  In the section: Low-density lipoprotein cholesterol (LDL-C) (in Materials and methods), we have *included the following*:  “LDL-C is given as mmol/L and can be converted to mg/dL by multiplying with 38.6.”  We have added the LDL-C values in mg/dL (in brackets) in the abstract, the manuscript, and in the figure and tables captions. | |
| #2. In the abstract, instead of reporting the proportions of patients who started LLT or intensive LLT or the proportions of patients who changed LLT intensity, it would be better to have the answers to the following questions: 1) which was the proportion of patients achieving the predefined LDL-C targets for those who started LLT within 90 -180 days?; 2) which was the proportion of patients achieving the LDL-C targets for those who started intensive LLT?; 3) how many patients who changed LLT intensity had their LLT up-titrated and how many had their LLT down-titrated? There were any difference between these two groups in terms of achievement of the predefined LDL-C targets? | The aim of the present paper is to examine trends and developments in LLT treatment patterns and LDL-C goal achievement in a national representative sample of individuals with an ASCVD diagnosis.  In a previous study (Kristensen et al., 2020 [reference 18 in the revised manuscript]) we examined the association between LLT intensity and LDL-C goal achievement  Changes in LLT intensity (unchanged, less, more) are presented in supplementary table S5. However, in the present study we have not examined the association with LDL-C goal achievement. | |
| #3. Some recent studies can be added to support the statement reported on page 5, lines 86-88, that a large proportions of high risk individuals do not achieve the recommended therapeutic targets for LDL-C and other lipid parameters in the real world practice. For example, the following references should be added: Cardiovasc Diabetol. 2021 Jul 16;20(1):144. doi: 10.1186/s12933-021-01338-y. + Atherosclerosis 2019 Jun;285:40-48. doi: 10.1016/j.atherosclerosis.2019.03.017 + Clin Cardiol. 2021 Nov;44(11):1575-1585. doi: 10.1002/clc.23725. | Thank you for the additional references, which are included in the revised manuscript.  *Additional references are included*:  12. Morieri ML, Perrone V, Veronesi C, Degli Esposti L, Andretta M, Plebani M, Fadini GP, Vigili de Kreutzenberg S, Avogaro A. Improving statin treatment strategies to reduce LDL-cholesterol: factors associated with targets' attainment in subjects with and without type 2 diabetes. Cardiovasc Diabetol. 2021;20(1):144.  13. Authors/Task Force Members; ESC Committee for Practice Guidelines (CPG); ESC National Cardiac Societies. 2019 ESC/EAS guidelines for the management of dyslipidaemias: Lipid modification to reduce cardiovascular risk. Atherosclerosis. 2019;290:140-205.  14. Gong Y, Li X, Ma X, Yu H, Li Y, Chen J, et al. Lipid goal attainment in post-acute coronary syndrome patients in China: Results from the 6-month real-world dyslipidemia international study II. *Clin Cardiol*. 2021; 44(11): 1575-1585. | |
| #4. Which were the main reasons for not starting LLT (or intensive LLT)? On page 10, lines 206-207, it is stated that proportion of individuals who initiated LLT within the first 90 days after index (event) increased from 48.6 to 56.0%; in other words, about half of the study population did not receive any LLT therapy after acute cardiovascular events. Which were the main reasons for these missed prescriptions in very high risk individuals? | This is a very important question, which we unfortunately do not have a clear answer to.  Information regarding LLT is based on prescription redemption. We have no data on actual issuing a prescription.  Reasons for these missed prescriptions could be a combination of: 1) hospitals not issuing the prescriptions, and 2) the individuals do not redeem the prescriptions. The hospital can dispense drugs for a few days to the patient at discharge (which is not recorded anywhere) with the message to contact the general practitioner for further treatment initiation. The individuals might refrain from using the drug due to concerns about side effects.  In the section Limitations (in the Discussion), we have *included the following sentence*:  “The health registers do not contain data that might explain the patterns identified. Thus, we had no information on reasons for a suboptimal LLT. The available prescription data regarding LLT are based on prescription redemption but without data on actual issuing a prescription.” | |
| #5. In line with the previous comment, it is not clear to me why not all patients with acute cardiovascular event did not have any LLT prescribed at hospital discharge and why they should start LLT after 90-180 days or later on. Please clarify which were the clinical indications, since they are not in line with recommendations from current international guidelines. | This is clearly not in line with recommendations from current international guidelines. However, other studies have shown the same tendency.  In continuation of the arguments above (#4), individuals might start LLT 90-180 days or later after discharge if the individuals were requested to contact the general practitioner to initiate LLT after discharge. | |
| #6. In line with comment #2, in the conclusive remarks more emphasis should be devoted to the proportions of patients achieving the recommended therapeutic targets after hospitalization for acute cardiovascular events, rather than on absolute proportions of patients starting LLT or changing LLT intensity. Given the fact that very low proportions of patients at high cardiovascular risk were treated for high cholesterol levels before the index event, it would be easy to expect a trend toward increase in LLT prescriptions after hospitalizations, mostly in the recent years. | We agree and have modified the conclusion accordingly.  The conclusion has been modified  *from*:  “In a large population-based cohort study of LDL-C goal achievement and LLT initiation in individuals with incident ASCVD, we found an increased proportion achieving LDL-C<1.8 mmol/L within the first 2 years after index date. The proportion of individuals initiating LLT within the year after index date increased, especially for intensive LLT initiation, although only one third were initiating intensive LLT in 2015. Although trends show an improvement in LDL-C goal achievement, a substantial number of the individuals at high risk of a CV event receive suboptimal treatment.”  *to:*  “In a large population-based cohort study of LDL-C goal achievement and LLT initiation in individuals with incident ASCVD, the proportion achieving LDL-C<1.8 mmol/L (70 mg/dL) within the first 2 years after index date increased from 40.5% in 2010 to 50.6% in 2015. The proportion of individuals initiating LLT within the year after index date increased, especially for intensive LLT initiation, although only one third were initiating intensive LLT in 2015. Although trends show an improvement in LDL-C goal achievement, 49.4% of the individuals at high risk of a CV event did not achieve the LDL-C goal within 2 years after ASCVD hospitalization.”  We accordingly modified the conclusion in the Abstract  *from*  “An increased proportion achieved LDL-C goal within the first 2 years after index date. The proportion of individuals initiating LLT within the first year after index date increased, especially for intensive LLT initiation, although only one third were initiating intensive LLT in 2015. Despite trends show an improvement in LDL-C goal achievement, a substantial number of the individuals at high risk of a CV event receive suboptimal treatment”  *to*:  “LDL-C goal achievement within the first 2 years after inclusion increased from 40.5% in 2010 to 50.6% in 2015. LLT initiation within the first year after inclusion increased, especially for intensive LLT, although only one third initiated intensive LLT in 2015. Despite trends show improvements in LDL-C goal achievement, 49.4% of individuals at high risk of a CV event did not achieve the LDL-C goal within 2 years after ASCVD hospitalization.” | |
|  |  | |

Reviewers' comments:

Reviewer's Responses to Questions

**Comments to the Author**

1. Is the manuscript technically sound, and do the data support the conclusions?

The manuscript must describe a technically sound piece of scientific research with data that supports the conclusions. Experiments must have been conducted rigorously, with appropriate controls, replication, and sample sizes. The conclusions must be drawn appropriately based on the data presented.

Reviewer #1: Yes

Reviewer #2: Yes

2. Has the statistical analysis been performed appropriately and rigorously?

Reviewer #1: Yes

Reviewer #2: Yes

3. Have the authors made all data underlying the findings in their manuscript fully available?

The [PLOS Data policy](https://eur03.safelinks.protection.outlook.com/?url=http%3A%2F%2Fwww.plosone.org%2Fstatic%2Fpolicies.action%23sharing&data=04%7C01%7Cake%40sdu.dk%7C4285abd1892a4f3f74a408d9f6181695%7C9a97c27db83e4694b35354bdbf18ab5b%7C0%7C0%7C637811406804912575%7CUnknown%7CTWFpbGZsb3d8eyJWIjoiMC4wLjAwMDAiLCJQIjoiV2luMzIiLCJBTiI6Ik1haWwiLCJXVCI6Mn0%3D%7C2000&sdata=CPm0WtxOUTnLjZMXMEy8yYOcfP6IgPT3EkXNS0WOAmM%3D&reserved=0) requires authors to make all data underlying the findings described in their manuscript fully available without restriction, with rare exception (please refer to the Data Availability Statement in the manuscript PDF file). The data should be provided as part of the manuscript or its supporting information, or deposited to a public repository. For example, in addition to summary statistics, the data points behind means, medians and variance measures should be available. If there are restrictions on publicly sharing data—e.g. participant privacy or use of data from a third party—those must be specified.

Reviewer #1: Yes

Reviewer #2: Yes

4. Is the manuscript presented in an intelligible fashion and written in standard English?

PLOS ONE does not copyedit accepted manuscripts, so the language in submitted articles must be clear, correct, and unambiguous. Any typographical or grammatical errors should be corrected at revision, so please note any specific errors here.

Reviewer #1: Yes

Reviewer #2: Yes

5. Review Comments to the Author

Please use the space provided to explain your answers to the questions above. You may also include additional comments for the author, including concerns about dual publication, research ethics, or publication ethics. (Please upload your review as an attachment if it exceeds 20,000 characters)

*See table above with reviewers’ comments and the response to each comment / AK Ersbøll*

6. PLOS authors have the option to publish the peer review history of their article ([what does this mean?](https://eur03.safelinks.protection.outlook.com/?url=https%3A%2F%2Fjournals.plos.org%2Fplosone%2Fs%2Feditorial-and-peer-review-process%23loc-peer-review-history&data=04%7C01%7Cake%40sdu.dk%7C4285abd1892a4f3f74a408d9f6181695%7C9a97c27db83e4694b35354bdbf18ab5b%7C0%7C0%7C637811406804912575%7CUnknown%7CTWFpbGZsb3d8eyJWIjoiMC4wLjAwMDAiLCJQIjoiV2luMzIiLCJBTiI6Ik1haWwiLCJXVCI6Mn0%3D%7C2000&sdata=r5lcNehjC2UrSsPiG%2FIVblx9vowrGA%2B2M17taLkDazI%3D&reserved=0)). If published, this will include your full peer review and any attached files.


If you choose “no”, your identity will remain anonymous but your review may still be made public.


**Do you want your identity to be public for this peer review?** For information about this choice, including consent withdrawal, please see our [Privacy Policy](https://eur03.safelinks.protection.outlook.com/?url=https%3A%2F%2Fwww.plos.org%2Fprivacy-policy&data=04%7C01%7Cake%40sdu.dk%7C4285abd1892a4f3f74a408d9f6181695%7C9a97c27db83e4694b35354bdbf18ab5b%7C0%7C0%7C637811406804912575%7CUnknown%7CTWFpbGZsb3d8eyJWIjoiMC4wLjAwMDAiLCJQIjoiV2luMzIiLCJBTiI6Ik1haWwiLCJXVCI6Mn0%3D%7C2000&sdata=6UuGVyq51sDxj99C89qOwhy9YWewTUD5xRr23RE%2Bcmo%3D&reserved=0).

Reviewer #1: No

Reviewer #2: No
