# Supplementary material for: Trends in low-density lipoprotein cholesterol goal achievement and changes in lipid-lowering therapy after incident atherosclerotic cardiovascular disease: Danish cohort study
Source: PLoS One. 2023 May 31;18(5):e0286376. doi: 10.1371/journal.pone.0286376 (PMC10231813; doi:10.1371/journal.pone.0286376)
Supplement: Attachment — Submitted filename: RevisionNoteR2.docx [file pone.0286376.s003.docx]

**PONE-D-21-35228 – second revision**

Trends in low-density lipoprotein cholesterol goal achievement and changes in lipid-lowering therapy after incident atherosclerotic cardiovascular disease: Danish cohort study.

| **REVISION NOTE** | | | |
| --- | --- | --- | --- |
|  | | | |
| **ADDITIONAL REQUIREMENTS** | **RESPONSE** | | |
| 1 A rebuttal letter that responds to each point raised by the academic editor and reviewer(s). You should upload this letter as a separate file labeled 'Response to Reviewers'. | A revision note is given below in this document | | |
| 2 A marked-up copy of your manuscript that highlights changes made to the original version. You should upload this as a separate file labeled 'Revised Manuscript with Track Changes'. | A version of the revised manuscript with track-changes is uploaded | | |
| 3 An unmarked version of your revised paper without tracked changes. You should upload this as a separate file labeled 'Manuscript' | A clean version of the revised manuscript is uploaded | | |
|  | | | |
| **REVIEWER #2 COMMENTS TO THE AUTHOR** | **RESPONSE** | | |
| Authors addressed some of the comments proposed and modified the manuscript accordingly. Please consider specific responses to the remaining issues:  Some recent studies can be added to support the statement reported on page 5, lines 86-88, that a large proportions of high risk individuals do not achieve the recommended therapeutic targets for LDL-C and other lipid parameters in the real world practice. For example, the following references should be added: Cardiovasc Diabetol. 2021 Jul 16;20(1):144. doi: 10.1186/s12933-021-01338-y. + Atherosclerosis 2019 Jun;285:40-48. doi: 10.1016/j.atherosclerosis.2019.03.017 + Clin Cardiol. 2021 Nov;44(11):1575-1585. doi: 10.1002/clc.23725. | Thank you very much for the review.  Thank you for the suggested papers to include.  The three suggested papers have been included on page 4, line 75 (revised manuscript). The list of References is updated accordingly. | | |
| Which were the main reasons for not starting LLT (or intensive LLT)? On page 10, lines 206-207, it is stated that proportion of individuals who initiated LLT within the first 90 days after index (event) increased from 48.6 to 56.0%; in other words, about half of the study population did not receive any LLT therapy after acute cardiovascular events. Which were the main reasons for these missed prescriptions in very high risk individuals? | The aim of the study is to examine trends in LDL-C goal achievement, trends in initiation in LLT therapy and changes in LLT intensity during the study period. Therefore, the suggested analysis is not considered within the scope of the manuscript.  However, we agree that it is a rather high proportion of the study population, who did not initiate LLT therapy. Therefore, we have performed a supplementary analysis of the association between sociodemographic and clinical characteristics and initiation of LLT therapy.  The statistical analysis is described in a new section: Supplementary analyses in Materials and Methods as:  “The second supplementary analysis examined sociodemographic and clinical characteristics associated with initiation of LLT therapy (moderate or intensive) between admission and 90 days after discharge among individuals with no LLT therapy before admission using a logistic regression model. LLT initiation was a binary outcome.”  The result of the analysis is presented in Supplementary Table S7 and described in a new section: Supplementary analyses in section Results as: “In the second supplementary analysis, sociodemographic and clinical characteristics associated with initiation of LLT therapy (moderate or intensive) among individuals with no LLT therapy before admission was examined. Odds for initiation of LLT was highest for individuals with AMI or IS as the qualifying ASCVD event at index date (AMI: OR=10.84 (95% CI: 9.41; 12.48); IS: OR=8.77 (95% CI: 7.65; 10.05)), younger age (<40 years: OR=3.95 (95% CI: 2.48; 6.28); 40-49 years: OR=2.85 (95% CI: 2.38; 3.41); 50-59 years: OR=1.51 (95% CI: 1.34; 1.71)), without comorbidity (not diabetes: OR=3.12 (95% CI: 2.75; 3.53); not CKD: OR=2.29 (95% CI: 1.78; 2.94)) and no LDL-C measurements before admission (OR=13.17 (95% CI: 11.18; 15.51)) (Table S7).” | | |
| In line with the previous comment, it is not clear to me why not all patients with acute cardiovascular event did not have any LLT prescribed at hospital discharge and why they should start LLT after 90-180 days or later on. Please clarify which were the clinical indications, since they are not in line with recommendations from current international guidelines. | We agree that it is an astonishing high proportion of the individuals who did not initiate LLT therapy between admission and 90 days after discharge. The supplementary analysis described above indicates that lack of LLT initiation is associated with other diagnoses/ procedures than AMI and IS, older age, comorbidity, and LDL-C measurements before admission.  The reason to include periods 90 days after discharge was to capture a potentially late initiation of LLT therapy, although guidelines recommend initiation of LLT therapy for all individuals at very high risk or with ASCVD.  In the description of Lipid-lowering therapy (LLT) in the Materials and Methods, the following description has been added:  “Initiation of LLT therapy was defined as having a prescription redemption of the above-described drugs before hospital admission (180 days before admission) and in periods after admission (admission-90 days after discharge, 91-180 days after discharge, 181-365 days after discharge). Initiation of LLT therapy later than 90 days after discharge was included to capture potentially late initiations, although clinical guidelines recommend initiation of individuals at very high risk or with ASCVD.” | | |
|  | | | |
| **REVIEWER #3 COMMENTS TO THE AUTHOR** | **RESPONSE** | | |
| While the revised paper showed the modest changes of lipid treatment during a period time in the past, three are still some issues remained to be clarified including those suggested in the previous review.  1. Since only one population is evaluated, it is not known if this is a usual condition or region-specific condition. It is well known that many factors including the budget administration, medicare system, national/regional treatment guidelines, the specialty of the doctors for patient care, and so on may significantly impact on the real world lipid treatment. The above information and data analysis may be required to improve the background, the understanding and the potential implication of the current findings. | Thank you very much for the review.  Unfortunately, we only have a population from a part of Denmark for the study, however, it is a representative sample of the Danish population (reference 29 in the manuscript). The reference compared sociodemographic and health related characteristics and found homogeneity between all five Danish regions. We included the following description in Materials and Methods, Inclusion and exclusion criteria:  “The population in the Region of Southern Denmark including Funen is a representative sample of the entire Danish population [29] regarding demographic and socioeconomic characteristics, healthcare utilization, and use of medication.”  Based on a suggestion from reviewer #2, we have included an analysis of the association between sociodemographic and clinical characteristics and initiation of LLT therapy.  The analysis and results are described above and included in the manuscript and as a supplementary table.  In the Introduction to the paper, we have included several references pointing in the same direction with a high proportion of individuals at very high risk or with ASCVD lacking initiation of LLT therapy.  Reference 29:  Henriksen DP, Rasmussen L, Hansen MR, Hallas J, Pottegård A. Comparison of the Five Danish Regions Regarding Demographic Characteristics, Healthcare Utilization, and Medication Use—A Descriptive Cross-Sectional Study. *PLoS ONE* 2015;**10**:e0140197. | | |
| 2. It seems all types of the patients including acute coronary syndrome, stable symptomatic CAD, and silent CAD were enrolled according to the definition of incident atherosclerotic cardiovascular disease in the current study. However, it is well known that the indication for lipid-lowering treatment especially the use of stratin could be quite different in case of acute coronary syndrome vs. stable CAD in clinical practice according to the contemporary guideline. It is critical to divide the patients according to acute coronary syndrome, stable symptomatic CAD, and silent CAD by their clinical presentation to see if there is difference in lipid lowering treatment as indicated. Hopefully such analysis may answer the questions of the reviewers and improve the understandings on the current data. | We agree with the reviewer’s comment regarding a mix of acute, stable, and silent cases. However, we do not have further clinical data to divide the individuals into the suggested categories.  To accommodate the suggested stratification, we have repeated the analysis of trends in initiation of LLT therapy where individuals are divided into two categories according to the qualifying ASCVD event (AMI and IS versus remaining diagnoses and/or procedures). The results are presented in a Supplementary table.  The statistical analysis is described in Supplementary analyses in Materials and Methods as:  “A third supplementary analysis of trends in initiation of LLT therapy was performed stratified by type of qualifying event of ASCVD (AMI and IS versus remaining diagnoses and/or procedures). This was performed to evaluate if initiation of LLT therapy varied depending on the severity of the ASCVD event.”  The result of the analysis is presented in Supplementary Table S8 and described in Supplementary analyses in section Results as:  “The third supplementary analysis examined trends in initiation of LLT therapy stratified by type of ASCVD event (AMI and IS versus remaining diagnoses and/or procedures). A remarkably higher proportion of individuals with AMI or IS initiated LLT therapy between admission and 90 days after discharge compared with the remaining ASCVD diagnoses (Table S8). Among individuals with AMI or IS, the proportion initiating LLT between admission and 90 days after discharge increased during the study period from 67.4% to 74.6%. Among individuals with other ASCVD diagnoses or procedures (SA, UA, PAD, CABG, PCI), the proportion initiating LLT between admission and 90 days after discharge also increased during the study period, however, from 23.1% to 25.0%.” | | |
| 3. As mentioned by the authors, there are significant portion of the patients still without (adequate) lipid lowering treatment in the current observations. It is very important to the potential implication of the current findings. Some additional analysis may be required to see if there is difference in the patient characteristics (age, gender, and some on), disease patterns, living areas, medical resources, economic status, the specialty of the in charge doctors, and so on between patients with and those without (adequate) lipid lowering treatment. It should be helpful to improve the future lipid control in such patients. | Based on a suggestion from reviewer #2, we have included an analysis of the association between sociodemographic and clinical characteristics and initiation of LLT therapy.  The analysis and results are described above and included in the manuscript and as a supplementary table. | | |
| 4. Further discussion to explain and interpret at the current findings is indicated, which may be possible if the above-mentioned data and statistical analysis are available. | The above-mentioned analysis has been included in the manuscript, results are presented and discussed in the revised version of the manuscript. | | |
|  | | | |
| **REVIEWER #4 COMMENTS TO THE AUTHOR** | **RESPONSE** | | |
| The authors have considered the reviewers’ suggestion and improved the paper accordingly. I’ve no further comments on it. | Thank you very much for reviewing the manuscript. | | |
|  | | | |
| **REVIEWER #5 COMMENTS TO THE AUTHOR** | **RESPONSE** | | |
| My major concern is that the 2019 revised guidelines recommend an even lower treatment goal (as LDL-C< 54 mg/dL) for individuals at very high risk. Thus, the results of the study may appear obsolete from this point of view. Obviously, they describe a trend that is independent from target values, but it could be very interesting to have some data on the initial and final proportion of individuals reaching this more ambitious target in this population, at least in form of supplementary table. Besides, this should be indicated as a major limitation of the study. | Thank you very much for reviewing the manuscript.  We agree with the reviewer’s concern.  In the original manuscript, we used the treatment goal for LDL-C at 1.8 mmol/L as this was the guideline level for the physicians to aim for during the study period. It cannot be ruled out, that the physicians would have suggested a more intensive LLT therapy with a lower guideline level.  However, to accommodate your and another reviewer’s comments, we have included this as a supplementary analysis.  The statistical analysis is described in Supplementary analyses in section Materials and Methods as:  “In the first supplementary analysis, the treatment goal for LDL-C for individuals at very high risk or with ASCVD was reduced to 1.4 mmol/L (55 mg/mL) as recommended in the 2019 revised guidelines [8]. The descriptive analysis of development of LDL-C goal achievement in the study period was repeated with LDL-C treatment goal at 1.4 mmol/L.“  The result of the analysis is presented in Supplementary Table S6 and described in Supplementary analyses in section Results as:  “In the first supplementary analysis, the treatment goal for LDL-C for individuals at very high risk or with ASCVD was reduced to 1.4 mmol/L (55 mg/mL) as recommended in the 2019 revised guidelines [8]. Only 23.8-29.3% of the individuals achieved the goal within 730 days. It is further seen that LDL-C goal achievement within the first 180 days increased from 4.9% in 2010 to 9.4% in 2015 (Table S6).” | | |
| A procedure of coronary of angiography (CAG) is not an equivalent of ASCVD. Namely, no coronary plaques could be demonstrated. So, the inclusion of these subjects should be also indicated as a study limitation. | In the present study, CAG is not used as a definition of ASCVD on its own, but in combination with either SA or UA.  We agree with the reviewer’s comment regarding a mix of diagnoses and procedures, although guidelines recommend individuals with all diagnoses or coronary revascularization included in the present study to be at very high CV risk.  Based on a similar comment from Reviewer #3, we have repeated the analysis of trends in initiation of LLT therapy where individuals are divided into two categories according to the qualifying ASCVD event (AMI and IS versus remaining diagnoses and/or procedures). The results are presented in a Supplementary table. | | |
| Minor concerns: According to the most recent guidelines, your population should be considered at very high risk and not at high risk. This should be specified. | We have changed the description of the population from “high risk” to “very high risk”, as suggested throughout the manuscript. | | |
| Definition of CKD should be specified. | The definition of CKD is given in **Supplementary Material *Table S1*** Codes for diagnoses, procedures, and drugs as: | | |
|  | Chronic kidney disease | 581*-584* (ICD-8)  N02*-N08*, N11*-N14*, N158*-N160*, N162*-N165*, N168, N18*-N19*, Z940, Z992 E102, E112, E122, E132, E142, I12*-I13*, Q61*, M300, M313, M319, M321B, N26* (ICD-10) | The Danish National Patient Register  (ICD codes) |
|  |  | 94300, 94340, 5748*  BJFD2*, KKAS* | The Danish National Patient Register (procedure codes) |
|  |  | | |
| In table 1, the indicated percent for total single ASCVD is relative to type of ASCVD, whereas that during or before hospitalization is relative to the proportion between these periods. This may be confounding and may be specified with a note or in the table legend. The same is valid for the other variables (calendar year, age group, and so on). | Thank you very much.  A note has been included in the table text as follows:  “Percentages are given by rows, except for Total where percentages are given within the column.” | | |
| I think that the note on lipid measurements within 18 months before ASCVD hospitalization is e and not a; besides, notes b, c and e are indicated in the legend but not in the main text of the table. | Thank you very much, it has been changed to “e”.  A description of the footnotes a-e is given below the table. | | |
| In the methods, you affirm to exclude patients with a previous event in the period 197-2009; this should be clearly specified in the study flow-chart. | Thank you very much. We have described that individuals with prevalent ASCVD were excluded, however, it was not shown in the study flow-chart.  The study flow-chart has been modified to include this initial step in data management. A total number of 39,608 individuals had a diagnosis of ASCVD in the period 1.1.1977-31.12.2015. We excluded 22,647 individuals with a diagnosis of ASCVD before start of the period (1.1.2010) as being prevalent, resulting in a population of 16,961 individuals.  The description of the study population in Results has been rephrased as:  “We identified a total of 39,608 individuals with a first-ever diagnosis of ASCVD in the period 1.1.1977-31.12.2015. We excluded 22,647 individuals with ASCVD before the study period, resulting in a total of 16,961 individuals with incident ASCVD in the study period 2010-2015. Among individuals who survived to discharge (15,396) 10% lack LDL-C measurements before and/or during hospitalization (Table S2). After exclusions for various reasons (Fig 1*,* Table S2), the final study population comprised 11,997 individuals who were discharged alive and with an LDL-C≥1.8 mmol/L (70 mg/dL) before or during ASCVD hospitalization.” | | |
| Discussion is clear but very synthetic; I think it may be enlarged focusing on the many study results. | We have extended the discussion with the new findings, and also included a section with implications of the findings in the present study.  **“Implications**  Our findings have clinical and public health implications. The ESC/EAS guidelines [8] consider individuals with the ASCVD diagnoses included in the present study to be at a very high CV risk. Furthermore, the guidelines recommend individuals at very high risk of a CV event to initiate LLT to reduce LDL-C (<1.4 mmol/L according to the 2019 guidelines). The findings in the present study indicate a need to focus on clinical practice of individuals with ASCVD to limit the risk of further CV events and mortality. A much larger proportion of individuals with UA, SA, PAD, or coronary revascularization (e.g., PCI and CABG) should initiate LLT. A focus is also needed on individuals at older age and individuals with comorbidities (CKD, DM) where only a low proportion of individuals initiates LLT.” | | |
|  | | | |
| **REVIEWER #6 COMMENTS TO THE AUTHOR** | **RESPONSE** | | |
| This paper reports data obtained in the years 2010 to 2015. In these years, the target for LDL-C in high-risk patients, who are included into this study, was set at 1.8 mmol/L.  As the authors indicate in their introduction, this target has been reduced to 1.4 mmol/L in the 2019 guideline. In the latter guidelines, an even lower target was recommended for patients who suffered from several ASCVD events within two years. This aspect has not been taken into consideration in this manuscript. | Thank you very much for reviewing the manuscript.  Reviewer #5 has a similar comment. We have included a supplementary analysis with LDL-C goal achievement changed to 1.4 mmol/L. See description above. | | |
| Authors looked at first-ever ASCVD cases only.  Lipoprotein(a) was not mentioned at all.  Data of this manuscript essentially only describe the treatment with statins and ezetimibe. The reviewer did not see numbers for the use of fibrates, bile acid sequestrants or nicotinic acid.  LLT was categorized by intensity groups which have been defined by the number of prescription redemptions. Individuals were followed for a maximum of two years with censoring at the end of the study period. | In the present study, focus has been on individuals with an incident ASCVD. The guidelines have specific recommendations for individuals with a second CV event, which is also relevant to focus on. We will consider this in a separate study.  In the present study, we did not have access to data regarding lipoprotein(a).  We have focused on statins and ezetimibe as two separate LLT treatments categories, whereas fibrates, bile acid sequestrants and nicotinic acid and derivates are combined into a group “Other non-statins” (see e.g., definition in supplementary Table S1). Due to a limited number of individuals treated with fibrates, bile acid sequestrants and nicotinic acid and derivates, we had to combine these drugs into one category.  LLT intensity was defined as a combination of at least two prescription redemptions, type of drug and dose. | | |
| The major finding of this study is that the proportion of individuals who achieved LDL-C<1.8 mmol/L (70 mg/dL) during the first 730 days after ASCVD hospitalization increased from 40.5% in 2010 to 50.6% in 2015. But unfortunately, as discussed by the authors in their responses to the comments of the previous two reviewers, no reason for this increase can be described. This is also discussed in the chapter on limitations of the study. | Reviewer #2 had a similar comment.  Therefore, we have performed a supplementary analysis of the association between sociodemographic and clinical characteristics and initiation of LLT therapy.  See the response above. | | |
| The scientific significance of the data given for different time periods (e g 181 – 365 days) is rather minimal. | The reason to include periods 90 days after discharge was to capture a potentially late initiation of LLT therapy, although guidelines recommend initiation of LLT therapy for all individuals at very high risk or with ASCVD.  In the description of Lipid-lowering therapy (LLT) in the Materials and Methods, the following description has been added:  “Initiation of LLT therapy was defined as having a prescription redemption of the above-described drugs before hospital admission (180 days before admission) and in periods after admission (admission-90 days after discharge, 91-180 days after discharge, 181-365 days after discharge). Initiation of LLT therapy later than 90 days after discharge was included to capture potentially late initiations, although clinical guidelines recommend initiation of individuals at very high risk or with ASCVD.” | | |
| It is astonishing to read the 10 % of the individuals with an incident ASCVD were excluded due to the lack of LDL-C measurements either before or during hospitalization | We agree in this astonishing observation, and have included a sentence in the Results section regarding the relatively large proportion of individuals with no LDL-C measurement before or during hospitalization:  “Among individuals who survived to discharge (15,396), 10% lack LDL-C measurements before and/or during hospitalization (Table S2).” | | |
| The figures are difficult to read – color is needed to differentiate the curves. | The figures have been improved and colors are used to differentiate the curves. | | |
|  | | | |
| **REVIEWER #7 COMMENTS TO THE AUTHOR** | **RESPONSE** | | |
| The authors correctly responded to the reviewers' comments and corrected the errors. The manuscript has improved readability and the message is now sharper in focus and clearer. | Thank you very much for reviewing the manuscript. | | |
|  | | | |
| **REVIEWER #8 COMMENTS TO THE AUTHOR** | **RESPONSE** | | |
| Appreciate all of your corrections. The paper reads well.  The big question ,of course, is why weren’t a higher proportion of patients able to reach a guideline recommended goal of LDL-C <1.8 mmol/L (70 mg/dl) within 2 years of a diagnosis of incident ASCVD. Hope that’s a source of further inquiry | Thank you very much for reviewing the manuscript.  We agree that it is a big and important question. We have included further analyses and hope the additional findings contribute to focus on specific groups where more focus is needed. | | |
