# Supplementary material for: Trends in low-density lipoprotein cholesterol goal achievement and changes in lipid-lowering therapy after incident atherosclerotic cardiovascular disease: Danish cohort study
Source: PLoS One. 2023 May 31;18(5):e0286376. doi: 10.1371/journal.pone.0286376 (PMC10231813; doi:10.1371/journal.pone.0286376)
Supplement: Attachment — Submitted filename: RevisionNoteR3.docx [file pone.0286376.s004.docx]

**PONE-D-21-35228 – third revision**

Trends in low-density lipoprotein cholesterol goal achievement and changes in lipid-lowering therapy after incident atherosclerotic cardiovascular disease: Danish cohort study.

| **REVISION NOTE** | |
| --- | --- |
|  | |
| **ADDITIONAL REQUIREMENTS** | **RESPONSE** |
| 1 A rebuttal letter that responds to each point raised by the academic editor and reviewer(s). You should upload this letter as a separate file labeled 'Response to Reviewers'. | A revision note is given below in this document |
| 2 A marked-up copy of your manuscript that highlights changes made to the original version. You should upload this as a separate file labeled 'Revised Manuscript with Track Changes'. | A version of the revised manuscript with track-changes is uploaded |
| 3 An unmarked version of your revised paper without tracked changes. You should upload this as a separate file labeled 'Manuscript' | A clean version of the revised manuscript is uploaded |
|  | |
| **REVIEWER #1 COMMENTS TO THE AUTHOR** | **RESPONSE** |
| The authors have satisfactorily responded to the problems which I raised in my previous review. The supplementary analyses clearly improved the quality of the manuscript. | Thank you very much. |
| The nature of this study based on prescription redemptions limits possible interpretations with respect to reasons for this low rate of achievement of target values for LDL-C. Physicians attitude towards the initiation of LLT cannot be evaluated in this way, but it may have had a major impact on the performance of LLT in a given patient. Data have been gathered about 10 years ago – since that time new developments of drugs were made, ASCVD is now more in the focus of the medical community. | We agree that a study as the present using register-based data cannot be used to evaluate physicians’ attitude towards the initiation of LLT.  The study can be used to identify the extent of the problem that only a low proportion of individuals with ASCVD initiate LLT. Furthermore, it illustrates the development in LLT initiation and LDL-C goal achievement. |
|  | |
| **REVIEWER #2 COMMENTS TO THE AUTHOR** | **RESPONSE** |
| This is a study that examined trends in lipid-lowering therapy (LLT) and low-density lipoprotein cholesterol (LDL-C) goal achievement among individuals with incident atherosclerotic cardiovascular disease (ASCVD) in Funen Island, Denmark, between 2010 and 2015.  The study found an overall improvement in LDL-C goal achievement and an increased initiation of LLT during the first year after ASCVD hospitalization, particularly in the initiation of intensive LLT. However, a large proportion of individuals did not achieve the LDL-C goal within 730 days after index date, and less than 30% of individuals achieved the revised LDL-C goal of 1.4 mmol/L within that same timeframe. The study also identified characteristics associated with higher odds for LLT initiation, including type of ASCVD event, younger age, no comorbidities, and no LDL-C measurements before index date. The study's findings were generally consistent with previous studies that examined trends in LDL-C goal achievement and LLT initiation in populations at high risk of cardiovascular events.  Study Design: The study utilized a retrospective cohort design which is appropriate for investigating the relationship between exposure and outcome in large populations. The study design enabled the researchers to establish temporal relationships between LDL-C goal achievement and LLT initiation, and ASCVD hospitalization.  Data Sources: The study utilized several sources of data including the Danish National Patient Register, the Danish National Prescription Registry, and the Danish Civil Registration System. These are reliable and validated sources of data, and their use strengthens the validity of the study.  Study Population: The study population was individuals with incident ASCVD on Funen Island, Denmark. The sample size was large (n=11,997), and the sample was representative of the general Danish population. However, the study may not be generalizable to populations outside Denmark.  Exposure and Outcome Measures: The study utilized LDL-C measurements as the primary exposure and LLT prescription redemption as a secondary exposure. The outcome measures were LDL-C goal achievement and LLT initiation. The use of these measures is consistent with current guidelines and strengthens the validity of the study.  Statistical Analysis: The statistical analysis was rigorous and appropriate. The researchers utilized multivariable logistic regression models to determine the odds of achieving LDL-C goal and initiating LLT. They also utilized Kaplan-Meier curves to determine the time to LDL-C goal achievement and LLT initiation. These methods were appropriate and strengthened the validity of the study. | Thank you very much for the review of the manuscript.  We agree with the nice summary of the study given by the reviewer. |
| Strengths: The authors describe a few legitimate strengths in their paper, here are a few more.  1. Large sample size: The study had a large sample size, which increased the precision of the estimates.  2. Use of reliable data sources: The study utilized reliable and validated data sources which strengthened the validity of the study.  3. Appropriate statistical analysis: The statistical analysis was rigorous and appropriate, which strengthened the validity of the study.  4. Temporal relationships: The study design enabled the researchers to establish temporal relationships between LDL-C goal achievement, LLT initiation, and ASCVD hospitalization. | Thank you very much for the additional strengths of the study, which have been included in the section Strengths in the Discussion.  We have included “increased the precision of the parameter estimates” in the sentence regarding a large, representative sample:  “This population-based cohort study has several strengths including a large, representative sample of individuals with incident ASCVD at very high risk of a CV event. This limits the impact of selection bias [36], *increased the precision of the parameter estimates,* and ensures a high level of completeness and validity [30,31,34].”  We have included then following sentence:  “The data sources used in the present study (i.e., laboratory database, prescription and patient registers) are reliable and validated with a high degree of completeness and validity [30,31,33]. This strengthened the validity of the study and the findings.”  We have included the following sentence:  “Finally, appropriate statistical analyses were applied which also strengthened the validity of the study and the findings.”  The sentence  “Furthermore, we followed a cohort of individuals at individual-level up to two years after index date which is preferable compared to previous studies using repeated cross-sectional studies [24,26].”  has been modified to  “Furthermore, we followed a cohort of individuals at individual-level up to two years after index date which enabled us to establish temporal relationships between ASCVD hospitalization, LLT initiation and LDL-C goal achievement. This is preferable compared to previous studies using repeated cross-sectional studies [24,26].” |
| Weaknesses: The authors describe a few legitimate limitations in their paper, here are a few more.  1. Limited generalizability: The study was conducted on Funen Island, Denmark, which may limit the generalizability of the findings to other populations.    2. Exclusion criteria: The study excluded individuals with missing data which may have resulted in a selection bias. But there is no better alternative that the authors could easily do at this point, so this was the appropriate approach to follow.  3. Limited information on lifestyle factors: The study did not include information on lifestyle factors such as diet and exercise which may have influenced LDL-C goal achievement and LLT initiation.  4. No adjustment for confounding by indication: The study did not adjust for confounding by indication, which may have influenced the estimates. | Thank you very much for the additional weaknesses of the study. We have included the following description in the section Limitations in the Discussion:  We included “and increased the generalizability of the findings” the following sentence:  “Including all laboratories in Denmark would have strengthened the study *and increased the* *generalizability of the findings*.”  In addition to individuals excluded due to the different exclusion criteria, only 59 were excluded due to missing data for the variables used.  We included the following sentence:  “In addition to individuals excluded due to the different exclusion criteria, we excluded individuals with a missing or invalid personal identification number. However, it concerned a limited number of individuals (N=59) and is considered not to have any impact on the results.”  We included the following sentence:  “We had no information of health behavior (such as diet and physical activity) which may influence LLT initiation and LDL-C goal achievement. However, we included information about income in the second supplementary analysis of sociodemographic and clinical characteristics associated with LLT initiation. Income (as a measure of socioeconomic status) and health behavior are to some extent correlated.”  Confounding by indication is an important issue in pharmaco-epidemiological studies in general.  The type of ASCVD diagnosis (acute myocardial infarction and ischemic stroke (AMI/IS) versus other) can be considered as a proxy for indication for treatment. Therefore, development in LLT initiation during the study period has been stratified by type of ASCVD diagnosis in one of the supplementary analyses (Supplementary Material *Table S8)*. Hereby we think we have considered and handled confounding by indication. |
| Ways for the authors to improve their paper: (mostly in future work)  1. The authors could include information on lifestyle factors such as diet and exercise to determine their influence on LDL-C goal achievement and LLT initiation.  2. The authors could adjust for confounding by indication to increase the validity of the estimates.  3. The authors could conduct the study on a larger and more diverse population to increase the generalizability of the findings.  4. The authors could consider using other measures of exposure such as total cholesterol or HDL-C to determine their influence on LDL-C goal achievement and LLT initiation. | Thank you very much for the suggestions.  It could be very relevant and interesting to examine the health behavior in relation to LLT initiation and LDL-C goal achievement. Unfortunately, we do not have access to these variables for the study population. Socioeconomic status is to some extent correlated with health behavior like diet and physical activity. We have included income as a socioeconomic variable in the analysis of the association between sociodemographic and clinical characteristics and LLT initiation.  See comment above: we think we have considered confounding by indication by stratify the development in LLT initiation by type of ASCVD diagnosis in the supplementary analysis.  This is a very relevant issue for further research. In the section Implications in the Discussion, we have included the following recommendations:  “It is recommended to replicate the study with other and larger populations to examine the generalizability of the findings. Furthermore, it is recommended to repeat the study to monitor the development and evaluate improvement in LLT initiation and LDL-C goal achievement in the ASCVD population in order to limit the risk of a CV event.”  We have examined the influence of lipid measurements (LDL-C, HDL-C, total cholesterol, and triglycerides) on LLT initiation. The lipid measurements are included as categorical variables in three categories (lowest 25%, middle 50% and highest 25%).  In the section Supplementary analyses in Materials and Methods, we have included the following description for the second supplementary analysis:  “In this analysis, lipid measurements (LDL-C, HDL-C, total cholesterol (TC) and triglycerides) were included as categorical variables in three categories (lowest 25%, middle 50%, and highest 25%).”  The results of the analysis of the lipid measurements are included in Supplementary Table S7.  The results are described and included in the section Supplementary analyses in Results:  “Odds for initiation of LLT was high for individuals with a moderate to high LDL-C measurement (LDL-C at 2.4-3.8: OR=6.99 (95% CI: 6.13; 7.96) and LDL-C>3.8: OR=18.38 (95% CI: 15.73; 21.46)), and with a moderate to high total cholesterol, TC (TC at 4.4-59: OR=4.14 (95% CI: 3.69; 4.65) and TC>5.9: OR=9.75 (95% CI: 8.50; 11.18)).” |
|  | |
